# Safety and Immunogenicity of ChAd63/MVA Pfs25-IMX313 in a Phase I First-in-Human Trial

**DOI:** 10.3389/fimmu.2021.694759

**Published:** 2021-07-14

**Authors:** Hans de Graaf, Ruth O. Payne, Iona Taylor, Kazutoyo Miura, Carol A. Long, Sean C. Elias, Marija Zaric, Angela M. Minassian, Sarah E. Silk, Lee Li, Ian D. Poulton, Megan Baker, Simon J. Draper, Diane Gbesemete, Nathan J. Brendish, Filipa Martins, Arianna Marini, David Mekhaiel, Nick J. Edwards, Rachel Roberts, Johan Vekemans, Sarah Moyle, Saul N. Faust, Eleanor Berrie, Alison M. Lawrie, Fergal Hill, Adrian V. S. Hill, Sumi Biswas

**Affiliations:** ^1^ NIHR Clinical Research Facility, University Hospital Southampton NHS Foundation Trust and Faculty of Medicine, University of Southampton, Southampton, United Kingdom; ^2^ The Jenner Institute, University of Oxford, Oxford, United Kingdom; ^3^ Laboratory of Malaria and Vector Research, National Institute of Allergy and Infectious Diseases, National Institutes of Health, Rockville, MD, United States; ^4^ GSK Vaccines, Wavre, Belgium; ^5^ Clinical Biomanufacturing Facility, University of Oxford, Oxford, United Kingdom; ^6^ OSIVAX​​, Lyon, France

**Keywords:** Pfs25, malaria, transmission-blocking, IMX313, vaccine

## Abstract

**Background:**

Transmission blocking vaccines targeting the sexual-stages of the malaria parasite could play a major role to achieve elimination and eradication of malaria. The *Plasmodium falciparum* Pfs25 protein (Pfs25) is the most clinically advanced candidate sexual-stage antigen. IMX313, a complement inhibitor C4b-binding protein that forms heptamers with the antigen fused to it, improve antibody responses. This is the first time that viral vectors have been used to induce antibodies in humans against an antigen that is expressed only in the mosquito vector.

**Methods:**

Clinical trial looking at safety and immunogenicity of two recombinant viral vectored vaccines encoding Pfs25-IMX313 in healthy malaria-naive adults. Replication-deficient chimpanzee adenovirus serotype 63 (ChAd63) and the attenuated orthopoxvirus modified vaccinia virus Ankara (MVA), encoding Pfs25-IMX313, were delivered by the intramuscular route in a heterologous prime-boost regimen using an 8-week interval. Safety data and samples for immunogenicity assays were taken at various time-points.

**Results:**

The reactogenicity of the vaccines was similar to that seen in previous trials using the same viral vectors encoding other antigens. The vaccines were immunogenic and induced both antibody and T cell responses against Pfs25, but significant transmission reducing activity (TRA) was not observed in most volunteers by standard membrane feeding assay.

**Conclusion:**

Both vaccines were well tolerated and demonstrated a favorable safety profile in malaria-naive adults. However, the transmission reducing activity of the antibodies generated were weak, suggesting the need for an alternative vaccine formulation.

**Trial Registration:**

Clinicaltrials.gov NCT02532049.

## Introduction

It was estimated that in 2018, malaria killed 405 000 people most of whom were children aged under 5 years (1). The global community has made a powerful economic and humanitarian case for continued investment in the fight against malaria with the aim of defeating malaria within the next 15 years (2), having already reduced the global incidence of malaria by 30% and the malaria mortality rate by 47% between 2001 and 2013 (3). Transmission control is an essential component of malaria control and elimination (4, 5). The strategic goals for 2030, set out in the Malaria Vaccine Technology Roadmap, include the development of transmission blocking malaria vaccines (TBVs) (6). Unlikely traditional vaccines, TBVs do not provide direct protection to the vaccinated individual but instead reduce or eliminate disease transmission within a vaccinated community. Malaria TBVs target sexual stages of the parasite, which do not cause disease within the human host but are critical for completing it’s life cycle in the mosquito vector (7).

The *Plasmodium falciparum* Pfs25 protein (Pfs25) is a leading candidate antigen for a TBV (8, 9). Pfs25 is a sexual stage antigen of *Plasmodium falciparum* that is expressed on the surface of the zygote and ookinete forms of the parasite, where it is involved in ookinete formation, as well as a possible role in traversal of the mid-gut epithelium (10). As Pfs25 is not displayed with the human host, it has not been under the same level of immune pressure as other *Plasmodium* antigens, making it an attractive vaccine target. Pfs25 was identified as the target of highly effective transmission-blocking monoclonal antibodies which were shown to prevent the ookinete to oocyst transition in the mosquito mid-gut in several pre-clinical studies, thereby blocking transmission (11–13).

It has modest immunogenicity as a monomeric protein, but when conjugated to itself or to other carrier proteins the immunogenicity is substantially improved (14). A recombinant Pfs25 protein administered with adjuvant ISA 51 in human has been tested previously but this trial had to be terminated due to erythema nodosum reactions likely related to this specific antigen/adjuvant combination (15). In *ex vivo* models, such as the standard membrane-feeding assay (SMFA), mosquitoes are fed cultured gametocytes in the presence of whole serum or purified IgG. In this assay, vaccine-induced antibodies generated against candidate antigens have been shown to reduce the transmission capacity of the parasite, although this was carried out on the *P. vivax* homologue Pvs25 (16). In two other clinical trials, conjugation of Pfs25 to a recombinant detoxified ExoProtein A (EPA) from *Pseudomonas aeruginosa* and a plant produced virus-like-particle displaying Pfs25 induced antibodies that recognized native protein on the parasite surface and demonstrated modest but insufficient TRA (5, 17).

TRA demonstrated in a SMFA has been shown to correlate with antibody titer and with antibody avidity (5, 18). In addition, a correlation between level of antigen-specific antibodies and their blocking efficacy was demonstrated in humans, as approximately 1000 units (95% Confidence Interval, 683–1565 units) of antibody were required to reach a 50% reduction in oocyst density (15, 16). Moreover, two studies demonstrated that concentration of 50-80 ug/mL of anti-Pfs25-specific antibodies in human serum was needed to provide significant oocyst reduction in the SMFA (5, 19).

IMX313 is a small protein domain that self-assembles into a nanoparticle with seven identical chains. The 55 amino acid sequence is a hybrid of the oligomerisation domains of two chicken C4b-binding proteins, both distant homologues of human Complement 4 binding protein (C4bp), simultaneously performing an adjuvant-like effect that improves antibody responses to the fused protein antigens (20). IMX313 has recently been tested in a Phase I clinical trial in Oxford as part of a candidate tuberculosis vaccine MVA 85A-IMX313 demonstrating favorable safety and immunogenicity profiles (21). Pre-clinical vaccine development demonstrated that mice immunized with the blood-stage malaria vaccine candidate MSP1_19_ fused to IMX313 were protected against challenge with a lethal dose of *P. yoelii* parasites (20). Similarly, in our murine study, Pfs25 fused to IMX313 and expressed in recombinant replication-deficient chimpanzee adenovirus serotype 63 (ChAd63) and an attenuated orthopoxvirus MVA viral vectors demonstrated significantly higher transmission-reducing activity than the vectors encoding monomeric Pfs25, rendering it a highly promising TBV candidate vaccine (22).

The design and administration of a recombinant replication–deficient adenovirus and an attenuated recombinant poxvirus vectors in a prime/boost regimen has been optimized over the last decade in preclinical models to induce antibodies in conjunction with T cell responses (23, 24), including studies of such vectors encoding sexual-stage malaria antigens (25, 26). These vectors, delivering antigens from *P. falciparum*, have now been shown to be safe and immunogenic, resulting in the development of both T cell and antibody responses in healthy European and American adult volunteers (26–32) as well as in African adults, children, and infants (33, 34).

Here, we report the safety and immunogenicity of ChAd63 and MVA vectors encoding Pfs25-IMX313. These vaccines were tested in an open-label dose-escalation Phase Ia study in healthy United Kingdom (UK) adults. We report that these vaccines demonstrate a favorable safety profile in malaria-naive adults and that anti Pfs25-specific antibodies, B cell and T cell responses can be induced by immunization in humans. However, vaccine-induced serum antibodies displayed weak transmission blocking activity, highlighting the need for further in-depth evaluation of the human immune responses induced by this vaccine candidate in order to inform improved design of future TBV vaccines.

## Materials and Methods

Detailed methods are provided in **Supplementary Methods**.

### ChAd63 and MVA Pfs25-IMX313 Vaccines

The design, production and preclinical testing of the viral vector vaccines used in this study have been reported previously (22). For the Pfs25-IMX313 constructs a 229 bp DNA fragment encoding the IMX313 domain was cloned at the C-terminus of Pfs25. The Pfs25-IMX313 insert was subcloned into the ChAd63 and MVA destination and shuttle vectors. ChAd63 Pfs25-IMX313 was manufactured under current Good Manufacturing Practice (cGMP) conditions by the Clinical Biomanufacturing Facility (CBF), University of Oxford, UK, and MVA Pfs25-IMX313 was manufactured under cGMP conditions by IDT Biologika GmbH, Germany, both as previously described (29, 35).

### Study Design and Approvals

This was a Phase I open-label, dose escalation, first-in-human, non-randomized trial of the viral vectored vaccines ChAd63 Pfs25-IMX313 and MVA Pfs25-IMX313 given in a prime-boost regimen with an eight week interval. The study was conducted at the Centre for Clinical Vaccinology and Tropical Medicine (CCVTM), University of Oxford, Oxford, UK and the NIHR-CRF University Hospital Southampton NHS Foundation Trust, Southampton, UK. The study received ethical approval from the Oxfordshire Research Ethics Committee A in the UK (REC reference 15/SC/0237). The study was also reviewed and approved by the UK Medicines and Healthcare products Regulatory Agency (MHRA, reference 21584/0344/001-0001). Volunteers signed written consent forms and consent was verified before each vaccination. The trial was registered on Clinicaltrials.gov (NCT02532049) and was conducted according to the principles of the current revision of the Declaration of Helsinki 2008 and in full conformity with the ICH guidelines for Good Clinical Practice (GCP). The primary endpoint of the study was to assess the safety of ChAd63 Pfs25-IMX313 and MVA Pfs25-IMX313, with secondary endpoints to assess immunogenicity and *ex-vivo* efficacy.

### Participants

Healthy, malaria-naive males and non-pregnant females aged 18-50 years were invited to participate in the study. Volunteers were recruited and vaccinated at the CCVTM, University of Oxford and at the NIHR CRF in Southampton. In total, twenty-six volunteers were enrolled, with twenty-four vaccinated as per protocol and twenty-two completing follow-up. A full list of inclusion and exclusion criteria is reported in **Supplementary Methods**.

### Safety Analysis

Following each vaccination, volunteers completed an electronic diary card for 28 days with any adverse event data. Data regarding SAEs were collected throughout the duration of the trial. Observations (heart rate, temperature and blood pressure) were taken at the clinic visits from the day of vaccination until the 28 day follow-up visit. Blood tests for exploratory immunology were taken at all visits except those occurring 2 days after each vaccination (i.e. days 2 and 58). Blood samples for safety (full blood count, liver function, urea and electrolytes) were carried out at screening, day 0, day 7 and day 28 for all groups, as well as on days 56, 63 and 84 for Groups 2B and 2C. Any solicited AEs occurring during the first 7 days following vaccination were defined as being at least possibly related to vaccination. The likely causality of all other AEs was assessed as described in the protocol and all AEs considered possibly, probably or definitely related to vaccination are reported (**Supplementary Table 1**). Further details on grading are provided in the Supplementary Material.

### Peptides and *Ex-Vivo* IFN-γ ELISPOT

Peptides spanning the Pfs25 and IMX313 inserts, as well as junctional regions and human C4bp (20mers overlapping by 10) were purchased from NEO Scientific and used for *ex vivo* IFN-γ ELISPOT (**Supplementary Table 2**). *Ex-vivo* IFN-γ ELISPOT was used to assess the kinetics and magnitude of the vaccine-induced T cell responses over time. Fresh PBMC were used in all assays using a previously described protocol (30), except that 50 µL/well Pfs25 and IMX313 peptide pools (**Supplementary Table 2**) (final concentration of each peptide 5 µg/mL) were added to triplicate test wells, 50 µL/well R10 and DMSO control were added to negative control wells, and 50 µL/well Staphylococcal enterotoxin B (SEB) (final concentration 0.02 µg/mL) plus phytohemagglutinin (PHA) (final concentration 10 µg/mL) was added to positive control wells. Spots were counted using an ELISPOT counter (Autoimmune Diagnostika (AID), Germany). Results are expressed as IFN-γ spot-forming units (SFU) per million PBMCs.

### Total IgG ELISAs

ELISAs were performed against full-length Pfs25 protein using standardized methodology as previously described (29, 30). Briefly ELISA plates were coated over-night with Pfs25 protein (2µg/mL, 50 µL per well). The plates were blocked with StartingBlock™ T20 (PBS) Blocking Buffer (ThermoFisher Scientific,UK) and the assay is performed by using a standard curve and internal controls from the reference serum. Unknown test serum samples from immunized volunteers are diluted and added in triplicate to the ELISA plate. After a two hour incubation period, the diluted sera were discarded, the plate was washed and a secondary polyclonal antibody against the γ–chain of human IgG conjugated to alkaline phosphatase (Sigma, UK) was added. After 1 hour incubation, followed by a wash step, the alkaline phosphatase substrate was added. The substrate is left to develop for 25 minutes and the absorbance at 405nm was read using a plate reader. A standard curve and Gen5 ELISA software v3.04 (BioTek, UK) was used to convert the OD405 of individual test samples into arbitrary units (AU). These responses in AU are reported in μg/mL following generation of a conversion factor by calibration-free concentration analysis (CFCA) as described in **Supplementary Materials and Methods**.

### Memory B Cell and ASC ELISPOT

mBC ELISPOT assays were performed as described in detail elsewhere (36). In brief, frozen PBMC were thawed and cultured with a polyclonal B cell stimulation mix containing Staphylococcus aureus Cowan strain Pansorbin cell ‘SAC’ (Calbiochem, Germany), the human TLR agonist CpG ODN-2006 (Invivogen, USA) and pokeweed mitogen ‘PWM’ (Sigma,UK) for 6 days, allowing mBC to differentiate into ASC. On day five of the experiment, ELISPOT plates were coated with Pfs25 produced in Drosophila melanogaster Schneider 2 (S2) cell lines or IMX313 produced in *E. coli* to measure the antigen-specific response and polyvalent goat-anti human IgG (Caltag Medsystems, UK) to measure the total IgG response. PBS coated wells were used as a negative control. On day six, cultured cells were transferred to the ELISPOT plate and incubated for 18-20 hours before developing with an anti-human IgG (γ-chain) antibody conjugated to alkaline phosphatase (Calbiochem, Germany) followed by a substrate buffer. Plates were counted using an AID ELISPOT plate reader. *Ex-vivo* ASC ELISPOT assays were performed exactly as above but using frozen PBMC directly prepared and added to the ELISPOT plate with no preceding 6 day culture.

### SMFA

The ability of vaccine-induced antibodies to block the development of *P. falciparum* strain NF54 was evaluated using the SMFA as previously described (37). The percentage of mature Stage V gametocytes was adjusted to 0.15% ± 0.05% and the male-female ratio is stable (almost always 1 male: 2–3 female). IgG were purified from each sample *via* Protein G affinity chromatography, and adjusted to a final concentration of 40 mg/mL in PBS. Gametocyte cultures were mixed with purified IgG at 15 mg/mL concentrations without human complement; the positive control mouse monoclonal antibody 4B7 was used at a concentration of 0.094 mg/mL. Gametocyte cultures mixed with samples were then fed to 4–6 day old starved female *Anopheles stephensi* (SDA 500) *via* a parafilm^®^ membrane. The mosquitoes were maintained at 26 °C and 80% relative humidity. After 8 days, midguts from twenty mosquitoes per group were dissected, oocysts counted and the number of infected mosquitoes recorded. Percent reduction in infection intensity was calculated relative to the respective control IgG tested in the same assay.

### Statistical Analysis

Data were analyzed using GraphPad Prism version 6.07 for Windows (GraphPad Software Inc., California, USA) and R (version 3.5.1). All tests were two-tailed and are described in the text. A value of *P* < 0.05 was considered significant.

## Results

### Study Design

**Figure 1:**
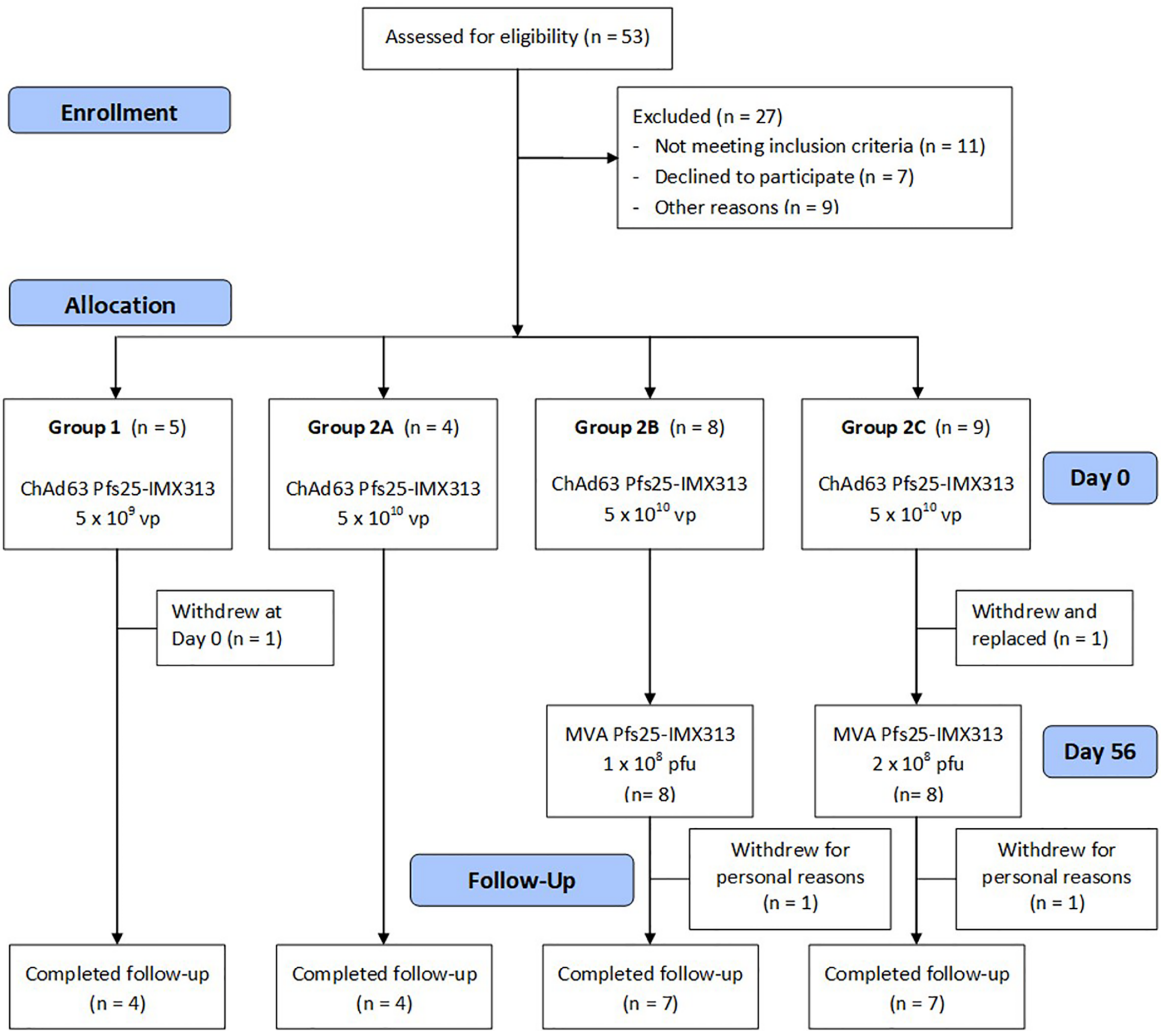
VAC062 flow chart of study design and volunteer recruitment. The VAC062 study took place between October 2015 and May 2017. All immunizations were administered intramuscularly (IM) with sequential vaccines administered into the deltoid muscle of the non-dominant arm.

Healthy UK adult volunteers were enrolled into the VAC062 trial to test the ChAd63/MVA Pfs25-IMX313 vaccine in an open-label, dose-escalation study design. Fifty-three UK adult volunteers were screened in total, of which twenty-six were enrolled (**Figure 1**). The first five volunteers were recruited to Group 1, however, one volunteer was withdrawn from Group 1 on the day of vaccination as they had been vaccinated with the low dose in error (the volunteer should have been enrolled into Group 2A and given the full dose). The remaining four volunteers were vaccinated with 5x10^9^vp of ChAd63 Pfs25-IMX313. The next four volunteers were recruited into Group 2A (vaccinated with 5x10^10^vp ChAd63 Pfs25-IMX313, eight volunteers to Groups 2B (vaccinated with 5x10^10^vp of ChAd63 Pfs25-IMX313 followed by 1x10^8^pfu of MVA Pfs25-IMX313) and the last nine volunteers into Group 2C (one volunteer from Group 2C withdrew and was replaced) (vaccinated with 5x10^10^vp of ChAd63 Pfs25-IMX313 followed by 2x10^8^pfu of MVA Pfs25-IMX313). 19 females and 7 males were enrolled into the study. The mean age of volunteers was 31 years (range 21 – 50 years), and 22 volunteers completed follow-up. Vaccinations began on 12^th^ October 2015 and all follow-up visits were completed by 25^th^ May 2017. All vaccinees received their ChAd63 Pfs25-IMX313 immunizations as scheduled. One volunteer in Group 2C withdrew before receiving their MVA Pfs25-IMX313 immunization (after the D28 follow-up visit) and was replaced, as permitted in the study protocol. There were two further withdrawals prior to the final D240 follow-up visit – one in Group 2B and one in Group 2C.

### ChAd63 and MVA Pfs25-IMX313 Vaccines Show a Favourable Safety Profile in Healthy UK Adult Volunteers

**Figure 2:**
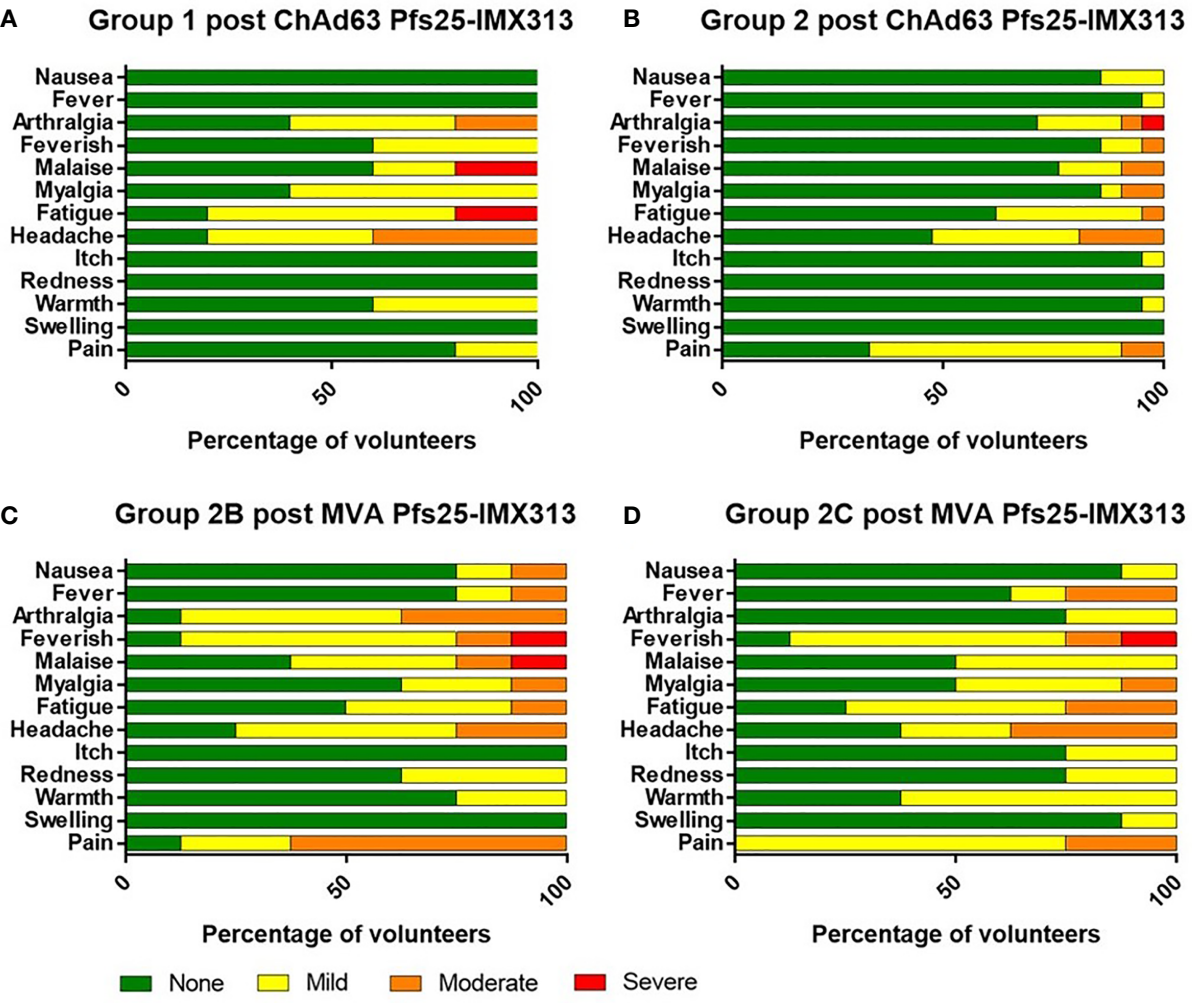
Solicited AEs following vaccination with ChAd63 and MVA Pfs25-IMX313. The solicited local and systemic adverse events (AEs) recorded for 7 days following ChAd63 Pfs25-IMX313 and MVA Pfs25-IMX313 are shown at the maximum severity reported by all volunteers. **(A)** Five volunteers received 5 × 10^9^ viral particles (vp) (Group 1), and **(B)** 21 received 5 × 10^10^ vp (Group 2) of ChAd63 Pfs25-IMX313. **(C)** Eight of the Group 2 volunteers went on to receive 1 × 10^8^ plaque-forming units (pfu) (Group 2B), or **(D)** Eight Group 2 volunteers received 2 × 10^8^ pfu (Group 2C) of MVA Pfs25-IMX313.

There were no serious adverse events (SAEs) or unexpected reactions during the course of the trial and no volunteers withdrew due to vaccine-related adverse events (AEs). The reactogenicity of the vaccines was similar to that seen in previous malaria vaccine trials using the same viral vectors at similar doses in healthy adults (28, 29, 32, 35), with the higher doses of both vaccines associated with an increased number of reported AEs (**Figure 2** and **Supplementary Table 1**).

### ChAd63 and MVA Pfs25-IMX313 Vaccination Induced IFN-γ T Cell Responses in Healthy UK Adult Volunteers

**Figure 3:**
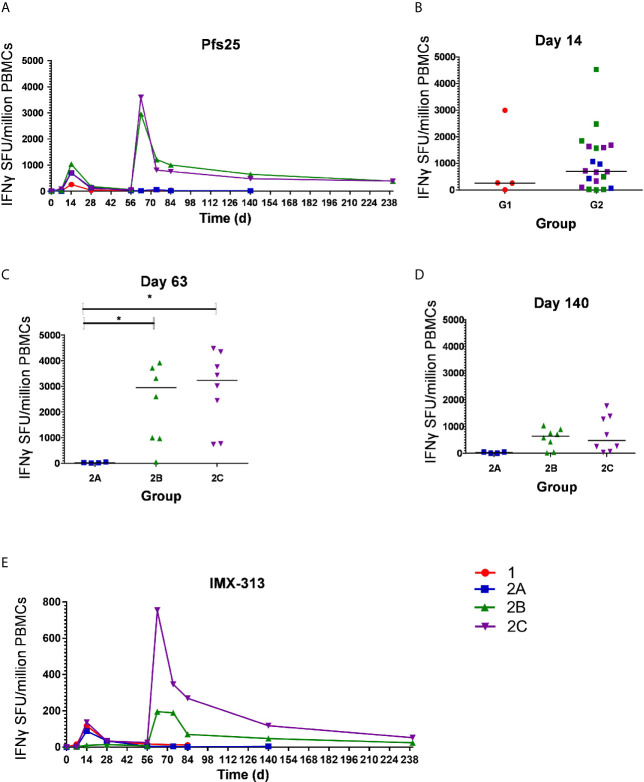
*Ex-vivo* IFN-γ T cell response against Pfs25 and IMX313. **(A)** Median *ex vivo* IFN-γ ELISPOT responses in PBMCs to the Pfs25 insert (summed response across all the individual peptide pools) are shown for all groups. Individual responses are shown in **Supplementary Figure 1**. Median and individual responses are shown at **(B)** day 14, **(C)** day 63, and **(D)** day 140. Symbols are coded according to group. *P < 0.05. Responses between Groups 1 (n = 4) and 2 (n = 20) at day 14, and between Groups 2B (n = 7) and 2C (n = 8) at day 140 were assessed by Mann-Whitney test **(B**, **D)**; responses between groups 2A (n = 4), 2B (n = 7), and 2C (n = 8) at day 63 were assessed by Kruskal-Wallis test with Dunn’s multiple comparison test **(C)**. **(E)** Median *ex vivo* IFN-γELISPOT responses in PBMCs to the IMX313 insert (summed response across a single peptide pool) shown for all groups. SFU, spot-forming units.

The kinetics and magnitude of the Pfs25-specific T cell response were assessed over time using *ex-vivo* IFN-γ ELISPOT (**Figure 3A**) assay following re-stimulation of PBMC with 20mer peptides overlapping by 10 amino acids (aa) (**Supplementary Table 2**) spanning the entire Pfs25 insert in the vaccines (22). Vaccination with ChAd63/MVA Pfs25-IMX313 induced antigen-specific T cell responses in all volunteers, with individual responses shown in **Supplementary Figures 1A–D** and median responses to the total vaccine insert shown for each group in **Figure 3A**. Following ChAd63 Pfs25-IMX313 prime, there was no significant difference between median responses in the lower dose Group 1 in comparison to Group 2 at the peak of the response on D14 (median 262 [range 5 – 2993] *vs* 702 [range 0 – 4533] spot forming units (SFU)/million PBMC in Groups 1 *versus* 2 respectively, n = 4 *vs* 20, P = 0.44 by Mann-Whitney test**) (** **Figure 3B**). Administration of MVA Pfs25-IMX313 significantly boosted these responses in all volunteers as measured one week later on D63 (Groups 2B and 2C *versus* 2A, Kruskal-Wallis test with Dunn’s multiple comparison test) (**Figure 3C**), reaching medians of 2953 [range 49–5988] and 3599 [range 736–4480] SFU/million PBMC in Groups 2B and 2C, respectively, *versus* 25 [range 11–49] SFU/million PBMC in Group 2A. However, there was no significant difference between the two groups who received the different doses of MVA Pfs25 (P=0.85, Mann-Whitney test). Following the peak at D63, responses contracted but were maintained above baseline at the end of the study period, with equivalently maintained responses at D140 in Group 2C as compared to Group 2B (**Figure 3D**). The T cell response to IMX313 was also measured as this was encoded in the viral-vectors (**Figure 3E**). Vaccine induced T cells to IMX313 followed a similar kinetic to Pfs25 responses but the magnitude of the response was much lower, likely due to being represented by a single peptide pool *vs* three for Pfs25.

At D0, D14 and D63 ELISPOT responses were also measured against C4bp, the human analogue of IMX313, to ensure no self-reactive T cells were induced. All responses were essentially negative as expected (**Supplementary Figure 2**).

### ChAd63 and MVA Pfs25-IMX313 Induce Serum Antibody Response in Healthy UK Adult Volunteers

**Figure 4:**
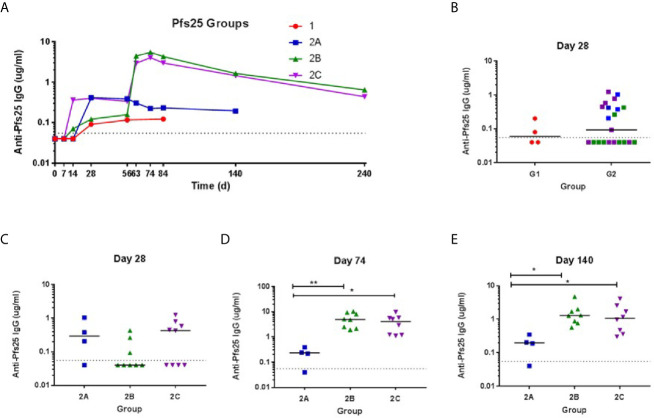
Serum antibody response against Pfs25. **(A)** Median anti–Pfs25 serum total IgG responses shown for all groups over time. Median and individual responses are shown at **(B, C)** day 28, **(D)** day 74, and **(E)** day 140. The horizontal dotted line indicates the limit of detection of the assay. Symbols are coded according to group. *P < 0.05, **P < 0.01. Responses between Groups 1 and 2 were assessed by Mann-Whitney test **(B)**. Responses in groups 2A (n = 4), 2B (n = 8), and 2C (n = 8) were assessed by Kruskal-Wallis test with Dunn’s multiple comparison test.

The kinetics and magnitude of the anti-Pfs25 serum IgG antibody response were assessed over time by ELISA against Pfs25 recombinant protein (**Figure 4**). Priming vaccination with 5 × 10^10^ vp ChAd63 Pfs25-IMX313 followed by MVA Pfs25-IMX313 boost induced antigen-specific IgG responses in all volunteers (Groups 2B and 2C), with individual responses shown in **Figures 4B–E** and median responses shown for each group in **Figure 4A**. Responses are reported in μg/ml following conversion of ELISA arbitrary units (AU) by calibration-free concentration analysis (CFCA) (**Supplementary Figure 3**). Following ChAd63 Pfs25-IMX313 prime with 5 × 10^9^ vp, 2 out of 4 volunteers seroconverted on D28, and after priming with 5 × 10^10^ vp 11 of 20 volunteers responded (median: 0.2992, range: 0.04-1.24 µg/ml, n = 20) (P = 0.41, Mann-Whitney test) (**Figure 4B**). The administration of MVA Pfs25-IMX313 boosted these responses as measured on D74 (peak at 10.24 µg/ml) (**Figure 4A**); this reached significance for Group 2B *vs.* 2A (P =0.008, Kruskal-Wallis test with Dunn’s multiple comparison test) and Group 2C *vs.* 2A (P = 0.035, Kruskal-Wallis test with Dunn’s multiple comparison test) (**Figure 4D**). Serum antibody responses decreased by D140 but were maintained above pre-boost levels. The responses in Group 2C and 2B was significantly higher than Group 2A at this time point (P= 0.0286 and P = 0.0075, respectively) Kruskal-Wallis test with Dunn’s multiple comparison test) (**Figure 4E**).

We also tested the sera from vaccinated individuals for reactivity to IMX313 in an ELISA. Anti-IMX313 IgGs were detectable at D28 post-vaccination in the sera from all immunization groups, and from D28, significantly increased at D74 for Groups 2A (P= 0.0052, Kruskal-Wallis test with Dunn’s multiple comparison test), 2B (P <0.000, Kruskal-Wallis test with Dunn’s multiple comparison test) and 2C (P=0.0002, Kruskal-Wallis test with Dunn’s multiple comparison test) (**Supplementary Figure 4A**). Generally, MVA boost did not increase antibody responses to IMX313. Only a marginally significant increase in anti-IMX313 antibody titers was observed on D74 for Group 2B *vs.* 2A (P = 0.03890, Kruskal-Wallis test with Dunn’s multiple comparison test), while no difference was evident between the groups on D84 (**Supplementary Figure 4B**). In addition, levels of the anti-IMX313 IgG antibodies positively correlated with the anti-Pfs25 antibody titers for all time points examined (D28, 74 and 84) (P<0.0001) (**Supplementary Figure 5**).

### ChAd63 and MVA Pfs25-IMX313 Induce B Cells in Healthy UK Adult Volunteers

**Figure 5:**
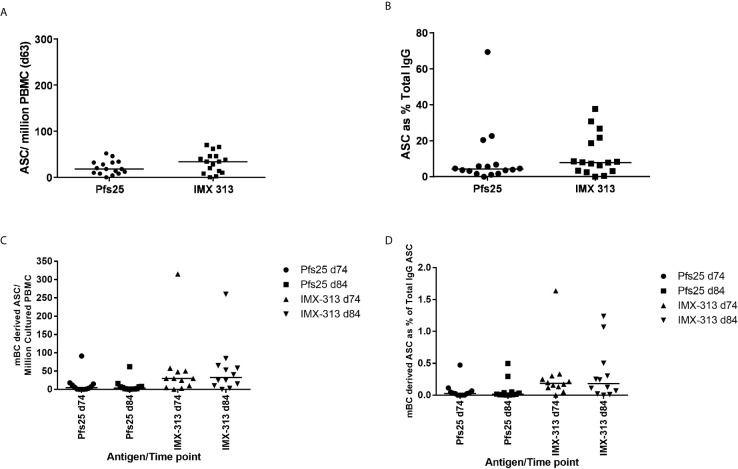
B cell response to vaccination. **(A)** Pfs25 and IMX313 –specific antibody-secreting cell (ASC) responses were assessed by ex-vivo ELISPOT using Pfs25 and IMX313 proteins and frozen PBMCs from the day 63 time point in Groups 2B and 2C. Individual and median responses are shown for each group and reported as Pfs25 and IMX313 –specific ASCs per million PBMCs used in the assay, or as **(B)** percentage of total number of IgG-secreting cells. **(C)** Pfs25 and IMX313 –specific memory B cell (mBC) responses were assessed by ELISPOT assay using Pfs25 and IMX313 proteins. Frozen PBMCs were thawed and underwent a 6-day polyclonal re-stimulation during which ASCs were derived from mBCs, before testing in the assay. Individual and median responses are shown from the day 74 and day 84 time point and are reported as mBC-derived Pfs25–specific ASCs per million cultured PBMCs or as **(D)** percentage of total number of IgG-secreting cells.

Pfs25 and IMX313 -specific antibody-secreting cells (ASC) responses were assessed by *ex-vivo* ELISPOT using frozen PBMC collected at the D63 visit for volunteers in Groups 2B and 2C. Median responses of 18 (Pfs25) *versus* 34 (IMX313) antigen-specific ASC per million PBMC were measured (**Figure 5A**). Percentage of total number of IgG-secreting cells was also assessed (**Figure 5B**). Previous studies have also reported that ASCs can be detected in peripheral blood shortly after the MVA boost when using the ChAd63/MVA regimen (31, 34).

Memory B cell (mBC) responses were also measured using an established cultured ELISPOT protocol whereby ASC numbers are measured using the same assay described above after the mBCs are stimulated for 6 days to convert them to ASCs. These were measured for volunteers in Groups 2B and 2C at the D84 time-point (4 weeks post-MVA boost) as this was the peak of the response identified in previous trials (32, 35) and D74 more recently identified as the likely peak of serum antibody responses. Responses are reported as number of mBC-derived Pfs25 and IMX313 -specific ASC per million cultured PBMC (**Figure 5C**), and as a % of total IgG-secreting ASC (**Figure 5D**). It was noted that IMX313 specific mBC responses were observed to be higher than Pfs25 responses at both time points though this was not significant (median 31 *vs* 5), and there was no significant difference in the responses between D74 and D84. Responses to Pfs25 were notably lower than those to another previously reported *Plasmodium falciparum* antigens delivered using the ChAd63/MVA vaccine regime (30).

### Functional Activity of Vaccine Induced Antibodies

**Figure 6:**
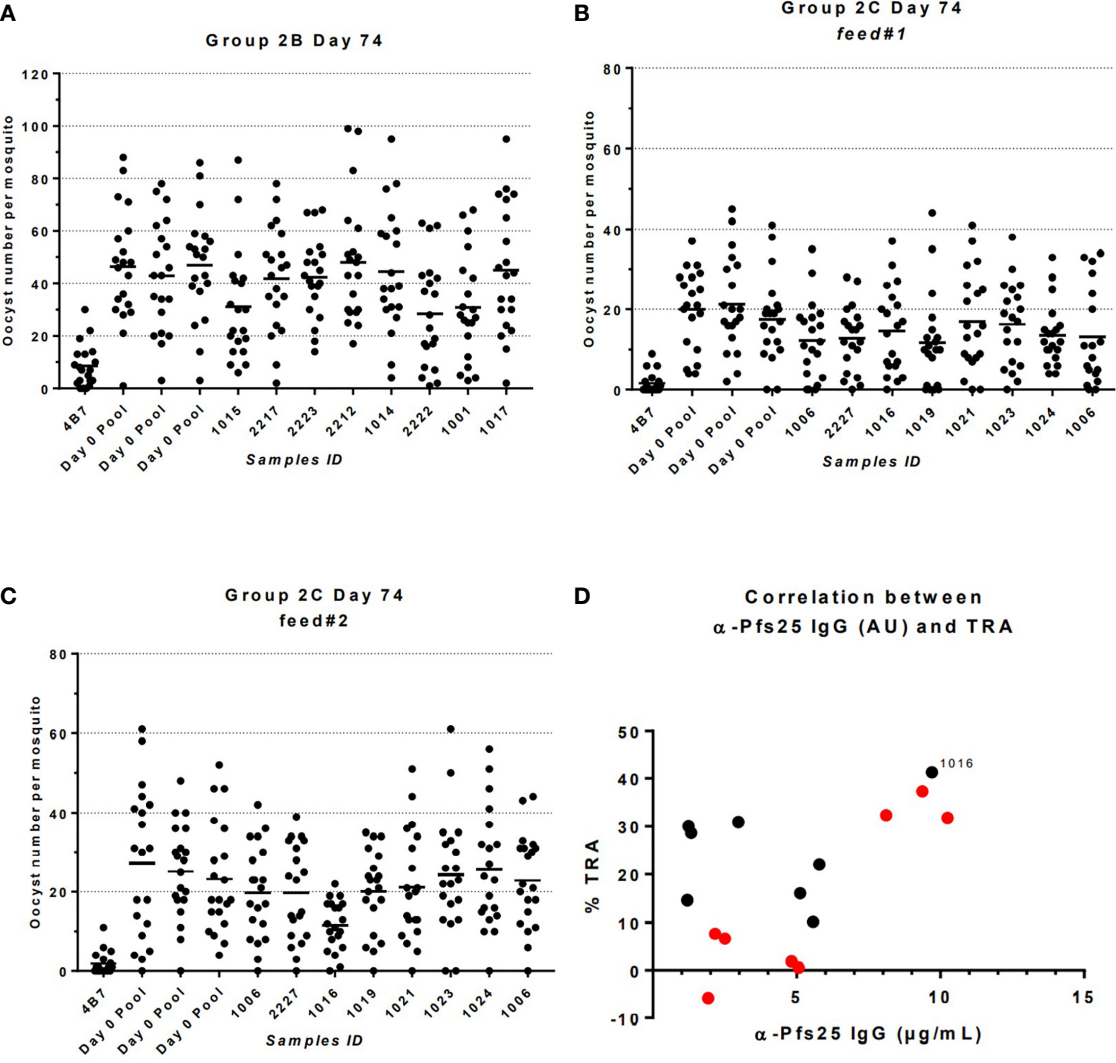
Transmission-reducing activity (TRA) in Groups 2B and 2C as measured by standardized membrane feeding assay. **(A)** Transmission-reducing activity of IgG from individuals of Group 2B. **(B, C)** Transmission-reducing activity of IgG from individuals of Group 2C. **(D)** Correlation between anti-Pfs25 specific IgG concentrations and TRA in individual of Groups 2B (in red) and 2C (in black); subject ID showing significant transmission-reducing activity is indicated. Total IgG was purified from D72 serum from volunteers vaccinated with ChAd63/MVA expressing Pfs25-IMX313. The purified IgG (15 mg/mL) was mixed with *P. falciparum* NF54 cultured gametocytes and fed to *A*. *stephensi* mosquitoes (n = 20 per test group) in SMFA. Midguts were dissected 7 days post-feeding. A pool of D0 vector immunized Group 2C volunteers was used as negative control.

The functional activity of the IgG generated in Groups 2B and 2C was tested using standard membrane feeding assay (**Figure 6**). Total IgG was purified from individual volunteers *via* Protein G affinity chromatography, mixed with *in vitro* cultured *P. falciparum* NF54 gametocytes, and fed to *Anopheles stephensi* mosquitoes (n = 20 per test sample) through a membrane feeder; mosquitoes were then dissected, and the number of oocysts counted. TRA was determined as the reduction in the number of oocysts compared to a negative control lacking protective antibody. SMFA with IgG at day 0 from Group 2C confirmed the lack of non-specific TRA (**Supplementary Table 3**) and a pool of these samples was then used as negative control for further assays. IgG at D74 from Groups 2B and 2C were tested at a concentration of 15 mg/ml in one or two independent feeds, respectively (**Figures 6A–C** and **Table 1**). Median TRA was 7.2% (range -5.8% to 37.3%) in Group 2B and 25.3% (range 10.2% to 41.3%) in Group 2C. None of the individual in Group 2B showed significant transmission-reducing activity in a single assay, while combined analysis of feed#1 and feed#2 for Group 2C showed that one out of eight individuals showed a weak, but significant, inhibition of oocyst intensity (subject ID 1016, 41.3% TRA, P = 0.043) (**Table 1**).

**Table 1:** Transmission-reducing activity of IgG from individuals of Groups 2B and 2C.

Group 2B	Mean OOC^A^	% TRA^B^	95% CI Low	95% CI High	*p* value*^c^*			
**Sample**								
**4B7^E^**	8.5							
**Day 0 Pool^F^**	46.5							
**Day 0 Pool^F^**	42.9							
**Day 0 Pool^F^**	47.0							
**1015**	31.1	31.7	-38.9	68.3	0.307			
**2217**	42.0	7.7	-96.2	57.5	0.801			
**2223**	42.4	6.7	-92.9	59.4	0.835			
**2212**	48.1	-5.8	-123.0	49.1	0.897			
**1014**	44.6	1.8	-106.4	56.7	0.911			
**2222**	28.5	37.3	-31.9	71.7	0.228			
**1001**	30.5	32.3	-47.8	70.5	0.324			
**1017**	45.2	0.5	-113.1	56.1	0.972			
**Group 2C**	**Feed#1**		**Feed#2**		**Cumulative analysis of the two feeds**			
	**MeanOOCA**	**% TRAB**	**Mean OOCA**	**% TRAB**	**% TRAB**	**95% CI Low**	**95% CI High**	***p* value^*c*^**
**Sample**								
**4B7^E^**	1.6		1.8^H^					
**Day 0 Pool^F^**	20.0		27.3					
**Day 0 Pool^F^**	21.3		25.2					
**Day 0 Pool^F^**	17.6		23.3					
**1006**	12.3^G^	37.5	19.8	21.7	30.0	-18.6	59.7	0.181
**2227**	12.8	34.7	19.7	21.9	28.6	-18.7	58.9	0.192
**1016**	14.7	25.0	11.6	54.0	41.3	1.7	66.2	*0.043*
**1019**	11.7	40.3	20.2	20.1	30.9	-13.1	61.6	0.147
**1021**	17.0	13.3	21.2	15.9	14.6	-45.7	50.8	0.565
**1023**	16.4	16.6	24.4	3.4	10.2	-51.1	48.8	0.652
**1024**	13.6	30.6	25.7	-1.7	16.0	-47.3	51.3	0.514
**1026**	13.2	32.8	22.9	9.4	22.0	-33.0	56.7	0.322

^A^Arithmetic mean of oocysts from 20 mosquitoes. ^B^Percent inhibition of mean oocyst intensity (95% CI). ^C^Two-tailed p values testing whether %TRA is significantly different from zero. ^E^Assay positive control. ^F^Pool of IgG at day 0 from Group 2C, tested in triplicate (total of 60 mosquitoes used for each feed, instead of 20). ^G^For 1006 in feed#1 only 18 mosquitoes had eggs. ^H^For 47B in feed#2 only 19 mosquitoes had eggs.

When the relationship between anti-Pfs25 IgG antibody concentration at day 74 of Groups 2B and 2C, and TRA was investigated a positive correlation was observed (Spearman r =0.5176; P = 0.0423). In particular, the subject ID 1016 in Group 2C showing significant inhibition is the one with the second highest levels of Pfs25-specific IgG antibodies.

## Discussion

Successful malaria vaccines that can contribute to malaria elimination will also need to have an impact on malaria transmission. In order to break the cycle of malaria transmission, TBVs will require mass administration to a broad population. Thus, developing a TBV with highly satisfactory safety profile and a formulation with minimally perceived risk are both mandatory for rapid clinical development. The results in our study showed the ChAd63/MVA Pfs25-IMX313 vaccine formulation demonstrated a favorable safety profile, being well tolerated at all dose levels, with most local and systemic adverse events being mild in severity, no apparent increase in adverse events with boost vaccination, and no participants withdrawn due to adverse events. Reactogenicity of the ChAd63/MVA Pfs25-IMX313 vector was similar to that seen consistently with the same doses of vectored vaccines encoding the *P. falciparum* pre-erythrocytic and erythrocytic malaria antigens (27–30, 32). Thus, our study supports the safety of clinical use of ChAd63/MVA delivery. In addition, confirming previous report in humans (21), no antibody or T cell-mediated cross-reactivity was detected in any of the study groups to the oligomerisation domain of human C4bp, which is likely due to the limited similarity between the IMX313 and human C4bp (22).

The ChAd63/MVA heterologous prime-boost immunization regimen induced both B and T cell responses and a modest level of anti-Pfs25 serum IgG titers. The ChAd63/MVA delivery platform has routinely been shown to induce a mixed antigen-specific CD4+/CD8+ T cell response in humans (38–40). Similar to data in other *P. falciparum* antigen studies in humans using these vaccine vectors (28, 29, 32, 41), the results presented here show that IFN-γ T cell responses were induced and peaked at median levels of greater than 2,000 SFU/million PBMCs following the MVA boost. The contribution of T cell responses to anti-Pfs25 immunity still remains unclear. However, a recent study, using Pfs25 encapsulated into synthetic vaccine particles as a vaccine in nonhuman primates, demonstrated that Pfs25-specific T cells may play a role in blocking malaria parasite transmission, by stimulating increased Ab avidity (18). In addition, another study demonstrated that the antibody response against Pfs25 was enhanced by carrier protein-dependent T-cell priming (40), suggesting that effector T cells may be important to induce the optimal antibody response against Pfs25.

In agreement with our preclinical data in mice (22), the ChAd63/MVA prime-boost regimen also induced anti-Pfs25 specific serum IgG antibody responses in healthy malaria-naive adult volunteers. Around 50% of participants had detectable antibody responses after the prime, with rapid boosting achieved after MVA Pfs25-IMX313 administration, when antigen-specific antibodies were induced in all volunteers. Boosting with the higher dose of MVA Pfs25-IMX313 (Group 2C) did not increase the antibody concentrations significantly, suggesting that beyond a certain vaccine dose threshold, the immunogenicity of MVA Pfs25-IMX313 does not improve. However, in another clinical study where malaria-naïve healthy adults received up to four doses of Pfs25-EPA conjugates formulated with Alhydrogel (5), the anti-Pfs25 antibody concentration was 10-fold higher compared to the level we observed in this study, suggesting that recombinant protein-in-adjuvant formulations may be superior over viral vectored vaccines in inducing antibody responses against mosquito stage malaria parasites.

We subsequently assessed the functional anti-parasitic antibody activity using the SMFA. These findings demonstrate weak transmission-reducing activity of antibodies in the serum of human volunteers vaccinated by ChAd63/MVA Pfs25-IMX313, when only one of sixteen vaccinated volunteers showed significant reduction in transmission activity. The TRA values of individual sera were low and highly variable, but weakly correlated with antibody concentrations measured by ELISA. However, oocyst counts in infected mosquitoes in the field are naturally much lower, in the range of 2–10 (42, 43), therefore the measured *ex-vivo* level of inhibition of oocyst development in the SMFA might be an under-estimation of the effect that could be achieved in the field challenge setting.

In contrast to the clinical trial data, our preclinical data in mice, confirmed that, in response to the same vaccines used in this clinical trial, high-titer functional anti-Pfs25 antibodies were induced, achieving extremely effective transmission-blocking activity. Consequently, information obtained from animal studies which assess efficacy of Pfs25 as a TBV candidate may not faithfully predict the results of human immunization. Notably in this regard, Cheru et al. demonstrated that the concentration of anti-Pfs25 human antibodies needed to inhibit 50 percent of oocyst development (IC50) in the membrane feeding assay varied among species, reporting that at least a 5-fold higher concentration of anti-Pfs25 human IgG antibodies (85.6µg/ml) was needed in comparison to mouse anti-Pfs25 IgG concentration (15.9 µg/ml) (19). In another Phase I clinical trial, following immunization with Pfs25-EPA/Alhydrogel^®^, the IC50 was estimated to be 57.2 μg/ml (5). In our study, a maximum of approximately 10µg/ml anti-Pfs25 antibodies were induced in the human serum, providing an explanation as to why transmission blocking activity was not observed by the SMFA in this clinical trial.

The lack of significant inhibition of oocyst intensity following ChAd63/MVA Pfs25-IMX313 makes further progression of this vaccination unlikely. Nevertheless, our findings highlight the need to explore the immunological basis for the inefficient TRA conferred by the ChAd63/MVA Pfs25-IMX313 vaccine, with a goal to improve future TBV immunization strategies. It will be of importance to further characterize the cellular and antibody responses, as it seems likely that differences in antibody function, avidity, IgG subclass profile, fine epitope specificity, as well as the potential requirement for tailored T-helper cell responses may all explain the weak inhibition of parasite growth mediated by this vaccine. With regards to other vaccine candidates, whilst a Phase I clinical trial of Pfs25 protein using Montanide ISA-51adjuvant had to be stopped due to safety issues (15), other Phase I studies in healthy adults (using either the plant-produced Pfs25 virus-like particles or Pfs25H-EPA conjugate vaccine with Alhydrogel^®^) suggested that a more immunogenic formulation is needed to effectively interrupt malaria transmission (5, 17). When Pfs25H-EPA in Alhydrogel^®^ was further tested in a naturally exposed population, it induced significant functional activity that blocked parasite transmission in a laboratory assay; however, this activity was only seen at peak titers after four vaccine doses, and antibody titers rapidly waned (44). Recent data from a clinical trial testing Pfs25 and Pfs230 conjugated to EPA reported that anti-Pfs230 antibodies have better functional activity than anti-Pfs25 antibodies (45). This highlights the need for testing additional antigens in clinical trials as pre-clinical animal studies are unable to underscore these differences.

Nevertheless, these prior trials in congruence with our study, suggest that transmission blocking immunity in humans using a Pfs25-based vaccine is feasible, but immunogenicity needs to be improved for this antigen, if 10x higher antibody concentrations are to be achieved, allowing this to be an effective constituent of a future multi-component malaria vaccine. New strategies to improve vaccine efficacy and well-tolerated formulations that consistently induce higher responses of longer duration are required, especially as Pfs25 is not naturally presented to the human immune system, natural boosting will not occur Alternate approaches, such as particulate delivery to improve immunogenicity and epitope-display, as well as alterations in schedule and dosing to improve qualitative and quantitative responses are being explored (46–48). Although efficacy is too low for this vaccine to have a role in public health in its present form, Pfs25 may be combined with other TBV, or pre-erythrocytic and/or erythrocytic vaccines which could permit use of more contemporary adjuvant formulations, with a potential to confer malaria protection.

## Data Availability Statement

The raw data supporting the conclusions of this article will be made available by the authors, without undue reservation.

## Ethics Statement

The studies involving human participants were reviewed and approved by the Oxfordshire Research Ethics Committee A in the UK (REC reference 15/SC/0237). The patients/participants provided their written informed consent to participate in this study.

## Author Contributions

Conceived and performed the experiments: HG, RP, IT, SE, KM, SS, IP, MB, MZ, AH, DG, NB, FM, and SF. Analyzed the data: HG, RP, AM, IT, SE, MZ, AM, SD, and SB. Contributed reagents/materials/analysis tools: DM, LL, JV, EB, FH, and SM. Project management: RR and AL. Wrote the paper: HG, RP, MZ, AM, DM, LL, and SB. All authors contributed to the article and approved the submitted version.

## Funding

This work was supported by the European Union Seventh Framework Programme (FP7/2007-2013) under the grant agreement for MultiMalVax (number 305282). The study was also supported in part by UK NIHR infrastructure through the NIHR Oxford Biomedical Research Centre (the views expressed are those of the authors and not necessarily those of the NHS, the NIHR or the Department of Health). The SMFA work was supported by the Intramural Research Program of NIAID, NIH and by PATH’s Malaria Vaccine Initiative. SD, AH, and SB are Jenner Investigators, and SD is also a Lister Institute Research Prize Fellow and a Wellcome Trust Senior Fellow [grant number 106917/Z/15/Z].

## Disclaimer

The opinions expressed herein are those of the authors and do not necessarily reflect the views and decisions of the World Health Organization. 

## Conflict of Interest

SB is a contributor in a patent application relating to multimerization technology. FH is named on patent applications relating to vaccines and immunization regimes. FH is an employee of OSIVAX, which owns rights to and is developing the IMX313 vaccine technology. AH and SD are named inventors on patent applications covering malaria vaccines and immunization regimens. JV was an employee of GlaxoSmithKline which has acquired the ChAd63 vector. AM has an immediate family member who is an inventor on patents relating to malaria vaccines and immunization regimens.

The remaining authors declare that the research was conducted in the absence of any commercial or financial relationships that could be construed as a potential conflict of interest.
