# Supplementary material for: Safety and Immunogenicity of ChAd63/MVA Pfs25-IMX313 in a Phase I First-in-Human Trial
Source: Front Immunol. 2021 Jul 14;12:694759. doi: 10.3389/fimmu.2021.694759 (PMC8318801; doi:10.3389/fimmu.2021.694759)
Supplement: Supplementary file 1 [file DataSheet_1.docx]

Supplementary Material

# Supplementary Materials and Methods

**Study Design**

The study was conducted in the UK at the Centre for Clinical Vaccinology and Tropical Medicine (CCVTM), University of Oxford, Oxford, and the NIHR CRF in Southampton. Healthy, malaria- naïve males and non-pregnant females aged 18-50 years were invited to participate in the study. Allocation to study groups occurred at screening based on sequential recruitment of groups and volunteer preference. For safety reasons the first volunteer who received a new vaccine dose was vaccinated alone and there was at least a 48 hour gap before subsequent volunteers were vaccinated. A further two volunteers could be vaccinated 48 hours after the first, and then at least another 48 hours gap had to elapse before the rest of the volunteers receiving that dose of vaccine could be vaccinated. Safety stopping and holding rules were used in this study to ensure participant safety, particularly given that this was a first-in-human dose escalation study, as detailed below.

**Group Holding Rules**

The study would have been put on hold if any of the following criteria were reached:

Solicited local adverse events:

- If more than 25% of doses of a vaccine were followed by the same Grade 3 local solicited adverse event beginning within 2 days after vaccination (day of vaccination and one subsequent day) and persisting at Grade 3 for > 48 h.

Solicited systemic adverse events:

- If more than 25% of doses of a vaccine were followed by the same Grade 3 systemic solicited adverse event beginning within 2 days after vaccination (day of vaccination and one subsequent day) and persisting at Grade 3 for > 48 h.

Unsolicited adverse events:

- If more than 25% of doses of a vaccine were followed by the same Grade 3 unsolicited adverse event (including the same laboratory adverse event) that was considered possibly, probably or definitely related to vaccination and persisted at Grade 3 for > 48 h. For the ChAd63 Pfs25-IMX313 vaccination, Groups 2A and 2B were considered as one group as they were enrolled simultaneously and received the same vaccine dose.

A serious adverse event considered possibly, probably or definitely related to vaccination, a death or a life-threatening reaction occurred.

**Individual Stopping Rules (applied to all vaccinated individuals)**

In addition to the above stated group holding rules, stopping rules for individual volunteers applied (i.e. indications to withdraw individuals from further vaccinations). Volunteers would have been withdrawn from further vaccinations if any of the events listed below occurred and were considered possibly, probably or definitely related to vaccination.

Local reactions:

- Injection site ulceration, abscess or necrosis.

Laboratory AEs:

- If the volunteer developed a Grade 3 laboratory adverse event considered possibly, probably or definitely related within 7 days after vaccination which persisted continuously at Grade 3 for > 72 h.

Systemic solicited adverse events:

- If the volunteer developed a Grade 3 systemic solicited adverse event considered possibly, probably or definitely related within 2 days after vaccination (day of vaccination and one subsequent day) which persisted continuously at Grade 3 for > 72 h.

Unsolicited adverse events:

- If the volunteer had a Grade 3 adverse event, which persisted continuously at Grade 3 for > 72 h.
- If the volunteer had a serious adverse event.
- If the volunteer had an acute allergic reaction or anaphylactic shock following the administration of the vaccine investigational product.

Safety reviews were carried out by the Local Safety Monitor (LSM) prior to each dose escalation, and no concerns were raised with any of the vaccine doses.

Participants in Groups 2B and 2C received the ChAd63 Pfs25-IMX313 and MVA Pfs25-IMX313 at days 0 and 56 (nominal study days are used throughout; a window period of ± 7 days was permitted in the protocol).

**Monitoring**

The LSM provided safety oversight, and Good Clinical Practice (GCP) compliance was independently monitored by the University of Oxford Clinical Trials and Research Governance (CTRG) Office.

**Inclusion and Exclusion Criteria**

A medical history and physical examination were conducted at the screening visit, as well as baseline blood tests including a full blood count; urea and electrolytes; liver function tests; and hepatitis B virus (HBV), hepatitis C virus (HCV) and human immunodeficiency virus (HIV) serology. Dipstick urinalysis for all volunteers and pregnancy testing for all female volunteers were conducted at screening. Pregnancy testing was also carried out prior to each vaccination. A full list of inclusion and exclusion criteria is shown below:

**Inclusion Criteria**

The volunteer had to satisfy all of the following criteria to be eligible for the study:

- Healthy adult aged 18 to 50 years;
- Able and willing (in the Investigator’s opinion) to comply with all study requirements;
- Willing to allow the discussion of their medical history with their GP;
- For females only, willingness to practice continuous effective contraception during the study and a negative pregnancy test on the days of screening and vaccination;
- Agreement to refrain from blood donation during the course of the study;
- Provision of written informed consent.

**Exclusion Criteria**

The volunteer could not enter the study if any of the following applied:

- Participation in another research study involving receipt of an investigational product in the 30 days preceding enrolment or during the study period;
- Prior receipt of an investigational malaria vaccine or any other investigational vaccine likely to impact on interpretation of the trial data;
- Administration of immunoglobulins and/or any blood products within the three months preceding vaccination;
- Any confirmed or suspected immunosuppressive or immunodeficient state, including HIV infection, asplenia, recurrent, severe infections and chronic (more than 14 days) immunosuppressant medication within 6 months preceding vaccination (inhaled and topical steroids were allowed);
- History of allergic disease or reactions likely to be exacerbated by any component of the vaccine, e.g. egg products;
- Any history of anaphylaxis in relation to vaccination;
- Pregnancy, lactation or willingness/intention to become pregnant during the study;
- History of cancer (except basal cell carcinoma of the skin and cervical carcinoma in situ);
- History of serious psychiatric condition likely to affect participation in the study;
- Any other serious chronic illness requiring hospital specialist supervision;
- Suspected or known current alcohol abuse as defined by an alcohol intake of greater than 42 units every week;
- Suspected or known injecting drug abuse in the 5 years preceding enrolment;
- Seropositive for hepatitis B surface antigen (HBsAg);
- Seropositive for hepatitis C virus (antibodies to HCV);
- History of clinical malaria (any species);
- Travel to a malaria endemic region during the study period or within the previous six months;
- Any clinically significant abnormal finding on screening biochemistry or haematology blood tests or urinalysis;
- Any other significant disease, disorder or finding which may significantly increase the risk to the volunteer because of participation in the study, affect the ability of the volunteer to participate in the study or impair interpretation of the study data;
- Inability of the study team to contact the volunteer’s GP to confirm medical history and safety to participate.

**Safety Analysis**

Following each vaccination, volunteers completed an electronic diary card for 28 days with any adverse event data. Participants were asked to record both solicited and unsolicited AEs. Data on solicited AEs were collected for 7 days after each vaccination, whereas details of any unsolicited AEs were collected for 28 days. Solicited AEs were those expected following an intramuscular (IM) vaccination and included local AEs (pain, erythema, warmth, swelling and itching) and systemic AEs (headache, malaise, myalgia, arthralgia, feverishness, nausea, fatigue, and measured fever). If these AEs occurred outside of the first seven days they were defined as unsolicited.

Volunteers graded all AEs as mild, moderate or severe:

GRADE 0: None.

GRADE 1: Transient or mild discomfort (< 48 h); no medical intervention/therapy required.

GRADE 2: Mild to moderate limitation in activity – some assistance may be needed; no or minimal medical intervention/therapy required.

GRADE 3: Marked limitation in activity, some assistance usually required; medical intervention/therapy required; hospitalization possible.

Adverse event data also included the results of the hematology (full blood count) and biochemistry (liver function tests, urea and electrolytes) carried out at all visits during the diary card period, except those occurring 2 days post-vaccination.

For each unsolicited AE, an assessment of the relationship of the AE to the study intervention(s) was undertaken. Alternative causes of the AE, such as the natural history of pre-existing medical conditions, concomitant therapy, other risk factors and the temporal relationship of the event to vaccination were considered. The likely causality of all unsolicited AEs was assessed as per the criteria below:

- **No Relationship**: No temporal relationship to study product *and* alternate aetiology (clinical state, environmental or other interventions); *and* does not follow known pattern of response to study product.
- **Unlikely:** Unlikely temporal relationship to study product *and* alternate aetiology likely (clinical state, environmental or other interventions) *and* does not follow known typical or plausible pattern of response to study product.
- **Possible:** Reasonable temporal relationship to study product; *or* event not readily produced by clinical state, environmental or other interventions; *or* similar pattern of response to that seen with other vaccines.
- **Probable:** Reasonable temporal relationship to study product; *and* event not readily produced by clinical state, environment, or other interventions *or* known pattern of response seen with other vaccines.
- **Definite:** Reasonable temporal relationship to study product; *and* event not readily produced by clinical state, environment, or other interventions; *and* known pattern of response seen with other vaccines.

All unsolicited AEs that were assessed as being possibly, probably or definitely related to either ChAd63 Pfs25-IMX313 are shown in Table S1. All unsolicited AEs that were assessed as being possibly, probably or definitely related to MVA Pfs25-IMX313 are shown in Table S1.

**ChAd63 and MVA Pfs25-IMX313 Vaccines**

For the Pfs25-IMX313 constructs a 229 bp DNA fragment encoding the IMX313 domain was cloned at the C-terminus of Pfs25. The Pfs25-IMX313 insert was subcloned into the ChAd63 and MVA destination and shuttle vectors. The recombinant viral vaccines were generated as described previously (1, 2). Basically, both transgene cassettes were inserted at the E1site of the E1/E3-deleted ChAd63 vector, under the control of the CMV immediate-early promoter. In the case of MVA, the transgene cassettes were inserted at the thymidine kinase (TK) locus with expression driven by the vaccinia P7.5 early/late promoter without any additional marker.

Manufacture of the drug substance for the ChAd63 (pool of individual lots of purified virus bulk harvest) is carried out in accordance with the requirements of cGMP by Clinical BioManufacturing Facility (CBF), Oxford.

The corresponding MVA vaccines were manufactured under cGMP conditions by IDT Biologika GmbH, Germany, as previously described (3).

Each vaccine lot underwent comprehensive quality control analysis to ensure that the purity, identity, and integrity of the virus met pre-defined specifications. Testing and specifications for the ChAd63 Drug Substance and Drug Product were done at Clinical BioManufacturing Facility at the Univesity of Oxford, in accordance with Ph. Eur. 5.14 monograph Gene transfer medicinal products for human use-Adenovectors for human use (01/2011).

**Study Design and Approvals**

Vaccination of volunteers was carried out at the CCVTM in Oxford and NIHR CRF in Southampton. The five volunteers in Group 1 were vaccinated with 5 x 10^9^ vp of ChAd63 Pfs25-IMX313, however, one volunteer was withdrawn from Group 1 on the day of vaccination as they had been vaccinated with the low dose in error (the volunteer should have been enrolled into Group 2A and given the full dose). Following a safety review, twelve volunteers (Groups 2A and 2B) were vaccinated with 5 x 10^10^ vp and eight of these (Group 2B) went on to receive MVA Pfs25-IMX313 1 x 10^8^ pfu eight weeks later. A further nine volunteers were enrolled into the final group (Group 2C), 1 volunteer from Group 2C withdrew after receiving ChAd63 Pfs25-IMX313 and was replaced. A safety review was carried out prior to the full dose MVA Pfs25-IMX313 vaccinations (2 x 10^8^ pfu) given eight weeks after ChAd63 Pfs25-IMX313 5 x 10^10^ vp.

Vaccination visits occurred on days 0 (all groups) and 56 (Groups 2B and 2C). Volunteers also attended follow-up visits on days 2, 7, 14, 28, 56 and 84 in Group 1, on days 2, 7, 14, 28, 56, 63, 84 and 140 in Group 2A and on days 2, 7, 14, 28, 56, 58, 63, 74, 84 and 140 in Groups 2B and 2C, with a final follow-up phone call for groups 1 and 2A on day 180 and on day 240 for Groups 2B and 2C. Nominal study days are reported throughout.

All vaccinations were given intramuscularly into the deltoid muscle, preferentially into the non- dominant arm. Blood tests for exploratory immunology were carried out at baseline (day 0) and at all physical visits after vaccination except days 2 and 58.

**Participants**

All volunteers signed written consent forms, and consent was confirmed before each vaccination. Allocation to study groups (Figure 1) occurred at screening based on sequential recruitment of groups and volunteer preference.

**Peripheral Blood Mononuclear Cell (PBMC) and Serum Preparation**

Blood samples were collected into lithium heparin-treated vacutainer blood collection systems (Becton Dickinson, UK). PBMC were isolated and used within 6 hours in fresh assays as previously described (4). Excess cells were frozen in foetal calf serum (FCS) containing 10% dimethyl sulfoxide (DMSO) and stored in liquid nitrogen. Plasma samples were stored at -80 °C. For serum preparation, untreated blood samples were stored at room temperature (RT) and then the clotted blood was centrifuged for 5 min (1000 *xg*). Serum was stored at -80 °C.

**Peptides**

Peptides for *ex-vivo* IFN-γ ELISPOT were purchased from NEO Scientific (Cambridge, MA, USA). The peptides, 20 aa in length and overlapping by 10 aa, covered the entire Pfs25 insert present in the viral vectored vaccines. Peptides were reconstituted in 100% DMSO at 50-200 mg/mL and combined into various pools for the ELISPOT assay.

**Proteins**

Recombinant Pfs25 protein for ELISA and B cell assays was produced from a stably transfected Drosophila melanogaster Schneider 2 (S2) cell line. Pfs25 sequence (GenBank accession no: AAN35500, from Alanine-22 to Threonine-193) with three potential N-linked glycosylation sites (112, 165, 187) mutated, was codon optimized for expression in insect cells (GeneArt® Life Technologies, Germany). Cell line generation and growth conditions have been previously described in detail (5, 6). Clarified supernatant from a 4-day batch culture of S2 cells was concentrated 15-20 fold and buffer exchanged using a TFF system fitted with Pellicon 3 Ultracel 3 kDa membrane (Merck Millipore, UK). Purification was performed on an AKTA Pure 25 system (GE Healthcare, UK), consisting of an affinity step with CaptureSelect™ C-tag column (Thermo Fisher Scientific, UK) and a polishing size exclusion chromatography (SEC) using Superdex 200 16/600 PG (GE Healthcare, UK) in 20 mM TrisHCl, 150 mM NaCl, pH 7.4 (TBS). Purified protein was quantified by Nanodrop (Thermo Fisher Scientific, UK) and stored at -80 °C until further use.

IMX313 protein used for ELISA was kindly provided by OSIVAX who owns rights to the IMX313 vaccine technology.

**Calibration-Free Concentration Analysis (CFCA)**

Anti-Pfs25 total IgG ELISA AU were converted to antigen-specific IgG concentration in µg/mL as follows. CFCA was performed with a method similar to that previously described (7-9), using a Biacore X100 instrument, a Biotin CAP chip, and X100 control and evaluation software (GE Lifesciences, UK). CFCA was performed using serum samples from subjects in Group 2 with a range of Pfs25-specific IgG antibody responses as assessed by ELISA. Each individual’s serum was diluted 1:20 in running buffer (7). Mass-transport limited binding conditions were obtained by capturing a minimum of 3000 response units (RU) of Pfs25 antigen on the active flow cell. The chip was regenerated with the manufacturer's supplied regeneration and CAP reagents (diluted 1:2 in HBS EP+) and fresh antigen (neat supernatant) prior to each application of antibody. Antigen-specific antibody binding was measured by double reference subtraction, firstly of binding to a flow cell coated only with the biotin capture reagent, and secondly of the binding of a day 0 serum sample (from the same volunteer) from that of the test sample. Initial rates of antigen-specific binding at 5 µL/min and 100 µL/min were measured and compared to permit measurement of concentration and the level of mass-transport limitation. Parameters (initial binding rate >0.3 RU/s at 5 µL/min flow rate and >0.13 for quality control (QC) ratio) recommended by the manufacturer were used for quality control of data. Initial binding rates were in the range 0.39 – 2.93 RU/s at 5 µL/min flow, and calculated QC ratios were all >0.3 (reflecting adequate mass transport limitation for concentration estimation). The binding model had a molecular weight of 150 kDa for IgG. The viscosity of the running buffer at 20 °C was 1.0562 mPa*s (measured using a densitometer equipped with a viscometer module) giving a measured diffusion coefficient of IgG at 20 °C in a solution with the viscosity of water, pH 7.4, of 4.8 x10^-11^ m^2^/s. The diffusion coefficient of IgG under the test conditions (25 °C) was therefore calculated to be 5.494 x 10^-11^ m^2^/s. The CFCA-measured Pfs25-specific antibody concentrations for each individual were analysed by linear regression with the corresponding total IgG ELISA AU data, with the slope of the line used to derive an AU-to-µg/mL conversion.

**References:**

1. Goodman AL, Blagborough AM, Biswas S, Wu YM, Hill AV, Sinden RE, et al. A Viral Vectored Prime-Boost Immunization Regime Targeting the Malaria Pfs25 Antigen Induces Transmission-Blocking Activity. *Plos One.* 2011;6(12).

2. Draper SJ, Moore AC, Goodman AL, Long CA, Holder AA, Gilbert SC, et al. Effective induction of high-titer antibodies by viral vector vaccines. *Nat Med.* 2008;14(8):819-21.

3. Sheehy SH, Duncan CJA, Elias SC, Collins KA, Ewer KJ, Spencer AJ, et al. Phase Ia Clinical Evaluation of the Plasmodium falciparum Blood-stage Antigen MSP1 in ChAd63 and MVA Vaccine Vectors. *Mol Ther.* 2011;19(12):2269-76.

4. Sheehy SH, Duncan CJA, Elias SC, Biswas S, Collins KA, O'Hara GA, et al. Phase Ia Clinical Evaluation of the Safety and Immunogenicity of the Plasmodium falciparum Blood-Stage Antigen AMA1 in ChAd63 and MVA Vaccine Vectors. *Plos One.* 2012;7(2).

5. Hjerrild KA, Jin J, Wright KE, Brown RE, Marshall JM, Labbe GM, et al. Production of full-length soluble Plasmodium falciparum RH5 protein vaccine using a Drosophila melanogaster Schneider 2 stable cell line system. *Sci Rep-Uk.* 2016;6.

6. Jin J, Hjerrild KA, Silk SE, Brown RE, Labbe GM, Marshall JM, et al. Accelerating the clinical development of protein-based vaccines for malaria by efficient purification using a four amino acid C-terminal 'C-tag'. *Int J Parasitol.* 2017;47(7):435-46.

7. Williams AR, Douglas AD, Miura K, Illingworth JJ, Choudhary P, Murungi LM, et al. Enhancing Blockade of Plasmodium falciparum Erythrocyte Invasion: Assessing Combinations of Antibodies against PfRH5 and Other Merozoite Antigens. *Plos Pathog.* 2012;8(11).

8. Hodgson SH, Choudhary P, Elias SC, Milne KH, Rampling TW, Biswas S, et al. Combining Viral Vectored and Protein-in-adjuvant Vaccines Against the Blood-stage Malaria Antigen AMA1: Report on a Phase 1a Clinical Trial. *Mol Ther.* 2014;22(12):2142-54.

9. Payne RO, Silk SE, Elias SC, Milne KH, Rawlinson TA, Llewellyn D, et al. Human vaccination against Plasmodium vivax Duffy-binding protein induces strain-transcending antibodies. *Jci Insight.* 2017;2(12).

## Supplementary Figures

| **Unsolicited AEs following ChAd63 Pfs25-IMX313** | | | | |
| --- | --- | --- | --- | --- |
| **Group** | **Subject ID** | **Timepoint(s)** | **Adverse event**  **(MedDRA LLT)** | **Maximum Severity** |
| 1 | MVT-0622205 | D5-D7 | Neck pain | 2 |
| 2A | MVT-0622202 | D1-D2 | Stiff neck | 1 |
| 2B | MVT-0621015 | D1-D2 | Chills | 2 |
|  | MVT-0622222 | D0-D1 | Chills | 1 |
|  | MVT-0622222 | D1-D2 | Decreased appetite | 1 |
|  | MVT-0621001 | D1-D2 | Photosensitivity | 1 |
| 2C | MVT-0621016 | D9-D11 | Injection site pain | 1 |
|  | MVT-0621019 | D12-D13 | Injection site pain | 1 |
|  | MVT-0621012 | D1-D2 | Dizziness | 1 |
|  | MVT-0621012 | D1-D2 | Chest tightness | 1 |
|  | MVT-0621024 | D0-D1 | Injection site pain | 1 |
| **Unsolicited AEs following MVA Pfs25-IMX313** | | | | |
| **Group** | **Subject ID** | **Timepoint(s)** | **Adverse event**  **(MedDRA LLT)** | **Severity** |
| 2B | MVT-0622212 | D1-D2 | Back ache | 1 |
|  | MVT-0621017 | D1-D4 | Dizziness on standing up | 2 |
| 2C | MVT-0621023 | D2-D6 | Erythema (distal to vaccination site) | 1 |

**Supplementary Table 1. Unsolicited AEs considered possibly, probably or definitely related to vaccination.** Adverse events as per MedDRA lower level term (LLT). Maximum reported severity shown. Time-point(s) = days post-vaccination

| **Name** | **Sequence** |  |  |
| --- | --- | --- | --- |
| Pfs25 1 | AKVTVDTVCKRGFLIQMSGH | P1 | Pfs25 |
| Pfs25 2 | RGFLIQMSGHLECKCENDLV |  |  |
| Pfs25 3 | LECKCENDLVLVNEETCEEK |  |  |
| Pfs25 4 | LVNEETCEEKVLKCDEKTVN |  |  |
| Pfs25 5 | VLKCDEKTVNKPCGDFSKCI |  |  |
| Pfs25 6 | KPCGDFSKCIKIDGNPVSYA | P2 |  |
| Pfs25 7 | KIDGNPVSYACKCNLGYDMV |  |  |
| Pfs25 8 | CKCNLGYDMVNNVCIPNECK |  |  |
| Pfs25 9 | NNVCIPNECKQVTCGNGKCI |  |  |
| Pfs25 10 | QVTCGNGKCILDTSNPVKTG |  |  |
| Pfs25 11 | LDTSNPVKTGVCSCNIGKVP | P3 |  |
| Pfs25 12 | VCSCNIGKVPNVQDQNKCSK |  |  |
| Pfs25 13 | NVQDQNKCSKDGETKCSLKC |  |  |
| Pfs25 14 | DGETKCSLKCLKEQETCKAV |  |  |
| Pfs25 15 | LKEQETCKAVDGIYKCDCKD |  |  |
| Pfs25 16 | DGIYKCDCKDGFIIDQESSI |  |  |
| Pfs25 17 | GFIIDQESSICT |  |  |
| IMX313 1 | GSKKQGDADVCGEVAYIQSV | P4 | IMX-313 |
| IMX313 2 | CGEVAYIQSVVSDCHVPTAE |  |  |
| IMX313 3 | VSDCHVPTAELRTLLEIRKL |  |  |
| IMX313 4 | LRTLLEIRKLFLEIQKLKVE |  |  |
| IMX313 5 | FLEIQKLKVELQGLSKE |  |  |
| J T-25 | IIDQESSICTGSKKQGDADV | P5 | Junctional |
| J25-IMX | QEIHARFRRSAKVTVDTVCK |  |  |
| hC4BP-1 | ETPEGCEQVLTGKRLMQCLP | P7 | hC4BP |
| hC4BP-2 | TGKRLMQCLPNPEDVKMALE |  |  |
| hC4BP-3 | NPEDVKMALEVYKLSLEIEQ |  |  |
| hC4BP-4 | VYKLSLEIEQLELQRDSARQ |  |  |
| hC4BP-5 | LELQRDSARQSTLDKEL |  |  |

**Supplementary Table 2. Pfs25 overlapping peptides for ELISPOT assays.** 20mer peptides overlapping by 10 aa were generated for the whole of the Pfs25 vaccine insert present in the ChAd63 and MVA. Peptide sequences are shown, and pools used in the ELISPOT assay indicated.

| Sample | Mean Ooc^A^ | % TRA^B^ | 95%CI Lo | 95%CI Hi | *p* value^C^ |
| --- | --- | --- | --- | --- | --- |
| 4B7^E^ | 1.0 |  |  |  |  |
| Normal mouse Ab^F^ | 10.4 |  |  |  |  |
| 1006 | 14.4 | -38.0 | -208.2 | 36.3 | 0.439 |
| 2227 | 11.1 | -6.7 | -138.1 | 52.5 | 0.883 |
| 1016 | 13.0 | -24.5 | -180.8 | 43.5 | 0.592 |
| 1019 | 16.6 | -59.1 | -280.4 | 36.0 | 0.311 |
| 1021 | 7.9 | 24.5 | -81.8 | 67.0 | 0.505 |
| 1023 | 7.0 | 32.7 | -48.0 | 70.8 | 0.319 |
| 1024 | 7.5 | 28.4 | -73.3 | 71.1 | 0.467 |
| 1026 | 17.9 | -71.6 | -295.2 | 24.2 | 0.206 |

**Supplementary Table 3. Transmission-reducing activity of IgG from individuals of Groups 2C at day 0.** **.** ^A^Arithmetic mean of oocysts from 20 mosquitoes. ^B^Percent inhibition of mean oocyst intensity in the 95% confidence interval (95% CI). ^C^Two-tailed p values testing whether %TRA is significantly different from zero.  ^F^Assay negative control. ^E^Assay positive control.

**Supplementary Figure 1. Individual ex-vivo IFN-γ ELISPOT data.**

ELISPOT responses to the Pfs25 insert (summed response across all the individual peptide pools) are shown over time following immunization in (A) Group 1 (n=4); (B) Group 2A (n=4); (C) Group 2B (n=8); and (D) Group 2C (n=8). Individual responses are shown for each volunteer.

**Supplementary Figure 2. Human C4bp ex-vivo IFN-γ ELISPOT data.**

ELISPOT responses to human C4bp are shown for D0 and D14 (Groups 1, 2A, 2B, 2C) and for D63 (Groups 2B, 2C). Responses below 20 sfu/million PBMC are considered below the detection threshold of the assay and are considered negative.

**Supplementary Figure 3. Calibration-free concentration analysis (CFCA).**

CFCA was performed using a Biacore X100 instrument in order to measure absolute (µg/ml) concentrations of Pfs25-specific antibody in serum samples from vaccinated subjects. (A) Binding at low sample flow rate (5 µl/min) is indicated in red while binding at high flow (100 µl/min) is indicated in green. Example data are shown for one test sample with the graph showing final double subtracted Pfs25-specific binding at the two flow rates. Sample binding to the non-Pfs25 coated reference flow cell (Fc1) was subtracted from the Pfs25-coated active flow cell (Fc2), followed by subtraction of volunteer-matched pre-immune d0 serum binding (also calculated as Fc2-Fc1). Coloured lines show the test sample data, and the black lines the fitted data generated by X100 evaluation software. The slopes of these solid lines were used to calculate antigen-specific antibody concentration in the test sample. (B) Correlation of ELISA AU for each sample and the Pfs25-specific IgG concentration measured by CFCA. Linear regression r^2^ value is shown; with the slope used to define the conversion factor between ELISA AU and antigen-specific IgG concentration in ng/ml.

**Supplementary Figure 4. Human IMX313 antibody response**

(A) D0, D28, D74 and D84 sera from Groups 1, 2A, 2B, 2C were tested for their antibody response against IMX313 protein by ELISA. (B) Individual and median responses for D28 (left panel), D74 (middle panel) and D84 sera (right panel) from groups 2A, 2B and 2C are shown. Kruskal Wallis test with Dunn’s multiple comparisons test was used to assess differences between the groups.

**Supplementary Figure 5. Correlation between anti-IMX313 and anti-Pfs25 antibody responses**

Data points show relationship between anti-IMX313 antibody titres detected in the sera of vaccinated individuals and corresponding anti-Pfs25 specific IgG concentrations (Spearman r = 0.91660; P =<0.0001). Values from D7, D28, D74 and D84 sera from Groups 2A, 2B and 2C are shown.
